# Designing and computational analyzing of chimeric long-lasting GLP-1 receptor agonists for type 2 diabetes

**DOI:** 10.1038/s41598-023-45185-1

**Published:** 2023-10-18

**Authors:** Maryam Ehsasatvatan, Bahram Baghban Kohnehrouz

**Affiliations:** Department of Plant Breeding and Biotechnology, Faculty of Agriculture, University of Tabriz, Tabriz, 51666 Iran

**Keywords:** Computational biology and bioinformatics, Dynamical systems

## Abstract

Glucagon-like peptide-1 (GLP-1) is an intestinally derived incretin that plays a vital role in engineering the biological circuit involved in treating type 2 diabetes. Exceedingly short half-life (1–2 min) of GLP-1 limits its therapeutic applicability, and the implication of its new variants is under question. Since albumin-binding DARPin as a mimetic molecule has been reported to increase the serum half-life of therapeutic compounds, the interaction of new variants of GLP-1 in fusion with DARPin needs to be examined against the GLP-1 receptor. This study was aimed to design stable and functional fusion proteins consisting of new protease-resistant GLP-1 mutants (mGLP1) genetically fused to DARPin as a critical step toward developing long-acting GLP-1 receptor agonists. The stability and solubility of the engineered fusion proteins were analyzed, and their secondary and tertiary structures were predicted and satisfactorily validated. Molecular dynamics simulation studies revealed that the predicted structures of engineered fusion proteins remained stable throughout the simulation. The relative binding affinity of the engineered fusion proteins' complex with human serum albumin and the GLP-1 receptor individually was assessed using molecular docking analyses. It revealed a higher affinity compared to the interaction of the individual GLP-1 and HSA-binding DARPin with the GLP-1 receptor and human serum albumin, respectively. The present study suggests that engineered fusion proteins can be used as a potential molecule in the treatment of type 2 diabetes, and this study provides insight into further experimental use of mimetic complexes as alternative molecules to be evaluated as new bio-breaks in the engineering of biological circuits in the treatment of type 2 diabetes.

## Introduction

Type 2 diabetes mellitus (T2DM) is a chronic, systemic metabolic disorder defined by persistent hyperglycemia and disturbances in carbohydrate, lipid, and protein metabolism due to a relative deficiency of insulin^1^. The global prevalence of this disease in humans has been estimated at 415 million adults in 2017 and is predicted to rise to 629 million by 2045^2^. Despite the availability of various T2DM treatments, such as diet, exercise, anti-diabetic medications, and subcutaneous insulin injection, glycemic control frequently fails due to inefficacy and poor patient compliance^3^.

Glucagon-like peptide-1 (GLP-1) and its analogs have emerged as promising medications for treating type 2 diabetes in recent years^4^. GLP-1 is a 30-residue incretin peptide hormone secreted into the bloodstream from the gut, primarily by distal ileum enteroendocrine L-cells in response to nutrient ingestion^5,6^. GLP-1 regulates blood glucose levels through several mechanisms, including stimulation of glucose-dependent secretion of insulin, suppression of pancreatic glucagon release, satiety enhancement, delayed stomach emptying, and reduction of energy intake^7–9^. These diverse mechanisms of action underpin the exceptional success of GLP-1 peptide analogues as diabetes and obesity treatments with little or no risk of hypoglycemia. However, the physiological half-life of GLP-1 in circulation is very short (less than 2 min), which can be attributed to both rapid proteolytic cleavage of the amide bond of alanine at position 8 (Ala8) in the N-terminus by ubiquitous dipeptidyl peptidase IV (DPP-IV), which results in the removal of the two N-terminal amino acids leading to the formation of a truncated inactive form^10,11^ and clearance by the kidney as well. As a result, significant efforts are being made to develop long-acting GLP-1R agonists, through the substitution of a susceptible amino acid in GLP-1 into a mutant to confer resistance to DPP-IV degradation^12^ and the significantly prolonging of GLP-1 half-life via the virtue of the neonatal Fc receptor (FcRn) recycling mechanism through binding to IgG-Fc^13^ or human serum albumin (HSA)^14^.

Fusion technology, which involves linking a target protein to a more stable protein, is one of the strategies used in protein-therapeutic optimization. Fusion proteins are a type of protein that links two or more distinct protein domains into a single molecule. Over the years, researchers have used recombinant DNA technology to mimic nature's strategy for creating artificial fusion proteins, such as the use of peptide or protein tags to enable affinity chromatography techniques for one-step protein purification^15,16^, fluorescent proteins as a biosensor for monitoring signaling molecules, or as a reporter in bioimaging^17^. Recently, synthetic biology applications have used artificial fusion proteins as novel protein switches^18^. Furthermore, synthetic antibody fragments and recombinant fusion proteins have developed as a new class of therapeutic medicines that can be used to address a wide range of issues in protein-drug development^19^. This approach has been proven to enhance protein-drug stability, activity, catalytic efficiency, and solubility. Furthermore, the fusion-protein method, which involves linking a target protein to a protein with a longer half-life, is employed to inhibit rapid renal clearance^20^.

Serum albumin binding has long been used in pharmacokinetic engineering to alter the pharmacokinetic features of small proteins. Human serum albumin (HSA) is a 66 kDa plasma protein with a long half-life of roughly 19–22 days in humans^21^. It is regularly utilized in fusion technology due to its prolonged half-life. Binding to HSA causes an increase in hydrodynamic volume, which slows renal clearance^13,14^. A further strategy to benefit from the extended plasma circulation of HSA is to derivatize GLP-1 with HSA-binding moieties. Several approaches have been described, including the use of natural albumin-binding proteins such as antibody fragments binding to serum albumin^22–24^, albumin-binding peptides^25^, the streptococcal protein G-derived albumin-binding domain^26,27^, and serum albumin-binding DARPin^28^.

Designed ankyrin repeat proteins are a promising class of small non-immunoglobulin binding proteins that bind target proteins with high affinity and specificity^29,30^. DARPin domains have desirable biophysical features, such as high thermal and thermodynamic stability, and can be expressed in high quantities in yeast, bacteria, and tobacco chloroplast expression systems^31–33^. Serum albumin-binding DARPin domains can be used to extend the terminal half-lives of next-generation protein therapeutics^28^. MP0250 is a tri-specific DARPin drug that contains one hepatocyte growth factor (HGF)-binding DARPin, one vascular endothelial growth factor (VEGF)-binding DARPin, and two HSA-binding DARPins to extend its half-life^34^. MP0250 has a half-life of about two weeks in humans^35^. This shows that DARPins with a high affinity for HSA can be employed as a universal carrier to extend their fusion half-life.

The current study aimed to develop long-acting protease-resistant GLP-1R agonists with the potential for administration in type 2 diabetes mellitus treatment by designing and computationally analyzing the fusion proteins of the native or protease-resistant mutant GLP-1 and an HSA-binding DARPin. An earlier study with albiglutide demonstrated that the presence of albumin directly next to GLP-1 could significantly reduce its activity^36^. Therefore, we engineered a chimeric protein by fusing GLP-1 to an HSA-binding DARPin with a high affinity for albumin that takes advantage of the extended plasma circulation of HSA to develop a long-acting variant of GLP-1. We incorporated a rigid helical linker between the GLP-1 molecule and the albumin-binding DARPin to keep protein moieties at a distance efficiently.

Since most of a protein's biological functions depend on its three-dimensional (3D) structure, appropriate folding, stability, and interaction between domains must be considered while creating multi-domain recombinant proteins. Unlike a single-domain protein, recombinant multi-domain proteins are more likely to misfold or assemble in an incorrect 3D shape because of the interaction between their distinct domains. Therefore, the in silico study of multi-domain proteins, followed by protein modeling and molecular dynamics (MD) simulations, is an essential stage in recombinant protein production efforts. We conducted a molecular modeling study of the engineered fusion proteins that could be used in T2DM treatment using the trRosetta protein structure prediction tool to obtain their 3D structures. The energy minimization and molecular dynamics simulation were done for the optimization of the models and monitoring their structural fluctuations. Also, the potential affinity of each moiety of the engineered fusion proteins against their specific targets was explored.

## Results

### Construct of the fusion proteins

**Figure 1:**
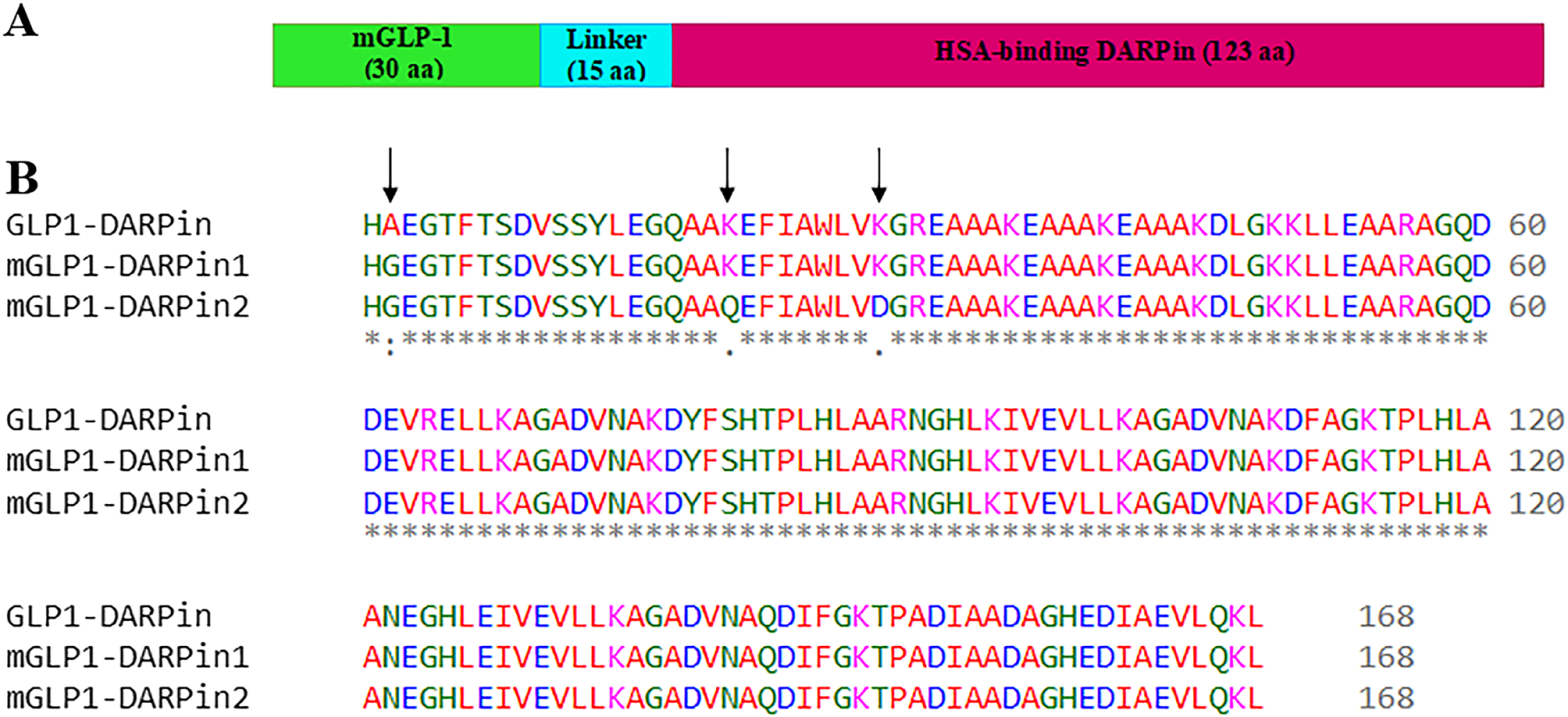
(**A**) Schematic model and (**B**) sequence alignment of engineered fusion proteins. Modified amino acids in GLP-1 were shown by the arrow.

Several flexible and rigid linkers were examined to select the top linker that maintains the functioning of the standard structure of the two domains of the fusion proteins (data not shown). Finally, the rigid helical (EAAAK)_3_ was used as the linker to maintain the spatial distance between the domains and their independent functions (Fig. 1A)^37^. The engineered fusion proteins became 168 amino acids in length, encoded by 504 nucleotides, including a native or modified GLP-1 (amino acids 1–30), a rigid helical linker (amino acids 31–45) as EAAAKEAAAKEAAAK, and amino acids 46–168 that belonged to HSA-binding DARPin. The multiple sequence alignment of modified proteins to demonstrate the differences between the native and modified GLP-1 moieties in engineered fusion proteins is shown in Fig. 1B.

### Primary structure analysis

The physicochemical parameters of the engineered fusion proteins were calculated from their primary structures using the ExPASy ProtParam tool and summarized in Table 1. The number of amino acids was 168 in all three fusion proteins, with an estimated molecular weight of around 17.5 kDa. The isoelectric point (pI) was calculated at 5.35 for nGLP1-DARPin and mGLP1-DARPin-1 and 5.01 for mGLP1-DARPin-2, indicating the dominance of amino acids with negative charges. Based on the computed instability index, all of the engineered fusion proteins were considered stable. The value of the aliphatic index, regarded as a positive factor for increased thermostability, was about 97 for all engineered fusion proteins. By considering the low instability index and relatively high aliphatic index, all engineered fusion proteins are classified as more thermally stable proteins. The calculated grand hydropathicity for fusion proteins exhibits a hydrophilicity pattern with better interaction with water. The solubility score based on the Protein-Sol web server was computed to be 0.796, 0.801, and 0.821 (cutoff: 0.45) for nGLP1-DARPin, mGLP1-DARPin-1, and mGLP1-DARPin-2, respectively. They indicate that the fusion proteins are soluble upon expression (Fig. S1). It is worthy of note that none of the engineered fusion proteins had toxic potential, according to the ToxDL server results (Table S1).

**Table 1:** Parameters calculated by ExPASy ProtParam tool.

Protein	Sequence length	Formula	Mw (Da)	TpI	− R	+ R	EC	II	AI	GRAVY
nGLP1-DARPin	168	C_768_H_1260_N_220_O_245_	17,711.98	5.35	28	20	8480	7.88	97.86	−0.179
mGLP1-DARPin-1	168	C_785_H_1258_N_220_O_245_	17,697.95	5.35	28	20	8480	6.82	97.26	−0.192
mGLP1-DARPin-2	168	C_782_H_1247_N_219_O_248_	17,684.82	5.01	29	18	8480	7.74	97.26	−0.188

*Mw* molecular weight, *TpI* theoretical isoelectric point, *−R* number of negative charged residues, *+R* number of positive charged residues, *EC* extinction coefficient at 280 nm, *II* instability index, *AI* aliphatic index, *GRAVY* grand average hydropathy.

### Secondary structure analysis

SOPMA and PORTER analysis of the secondary structure of the engineered fusion proteins revealed that they are predominantly composed of alpha-helices and random coils, and there was no significant beta-turn or extended strand. The rigid linker fragment within amino acids 31–45 was shown by helical structures (Figure S2).

### Tertiary structure prediction and evaluation

**Figure 2:**
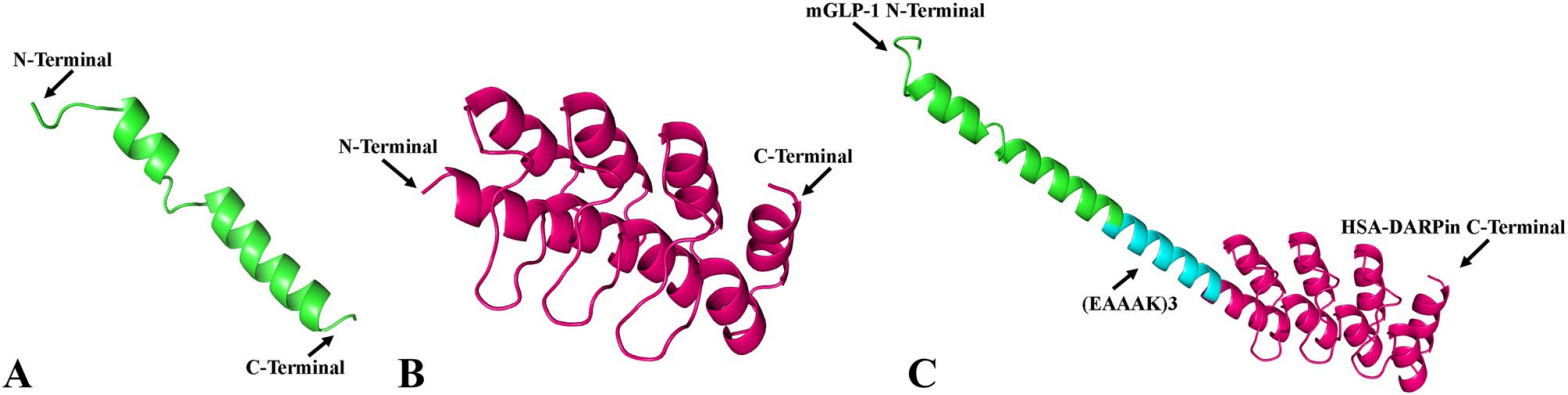
Three-dimensional structures of (**A**) GLP-1 (PDB id: 1D0R) and (**B**) HSA-binding DARPin (PDB id: 4GRG) from the RCBS PDB database; (**C**) trRosetta predicted model of the fusion protein containing GLP-1 (green), HSA-binding DARPin (magenta), and (EAAAK)_3_ rigid helical linker (cyan).

The prediction of the 3D structure was performed by the trRosetta online server. trRosetta is free for academic users and enables them to create excellent models of protein 3D structures using amino acid sequences. trRosetta's 3D structure prediction algorithm generates the five best models, with TM scores ranging from 0 to 1. The best model is the one with the highest TM score. The highest TM scores for predicted models of engineered fusion proteins were 0.928, 0.946, and 0.930 for nGLP1-DARPin, mGLP1-DARPin-1, and mGLP1-DARPin-2, respectively. The predicted models for engineered fusion proteins showed a protein with two separate domains connected through a small helical linker. The (AEEEK)_3_ rigid linker keeps domains apart and ensures a rigid separation of the individual domains of the fusion proteins. The NMR analysis of GLP-1 structures revealed two α-helix between residues 13–20 and 24–35 linked by a short linker region composed of residues 21–23, while the N-terminal residues 7–13 are unstructured (Fig. 2A)^38^. HSA-binding DARPin consists of two internal ankyrins with flanking constant N- and C-terminal capping repeats, each repeat forming a β-turn followed by two anti-parallel α-helix and an unstructured loop that binds to the β-turn of the next ankyrin repeat (Fig. 2B)^28^. As predicted by trRosetta, the fusion of HSA-binding DARPin to GLP-1 using a rigid helical linker does not alter the tertiary structure of the fusion partner, and each moiety of the fusion proteins will most likely retain their conformational properties and relevant biological functions as the native protein. The pre-simulated 3D structure of the mGLP1-DARPin-2 fusion protein, as an example, is shown in Fig. 2C.

**Figure 3:**
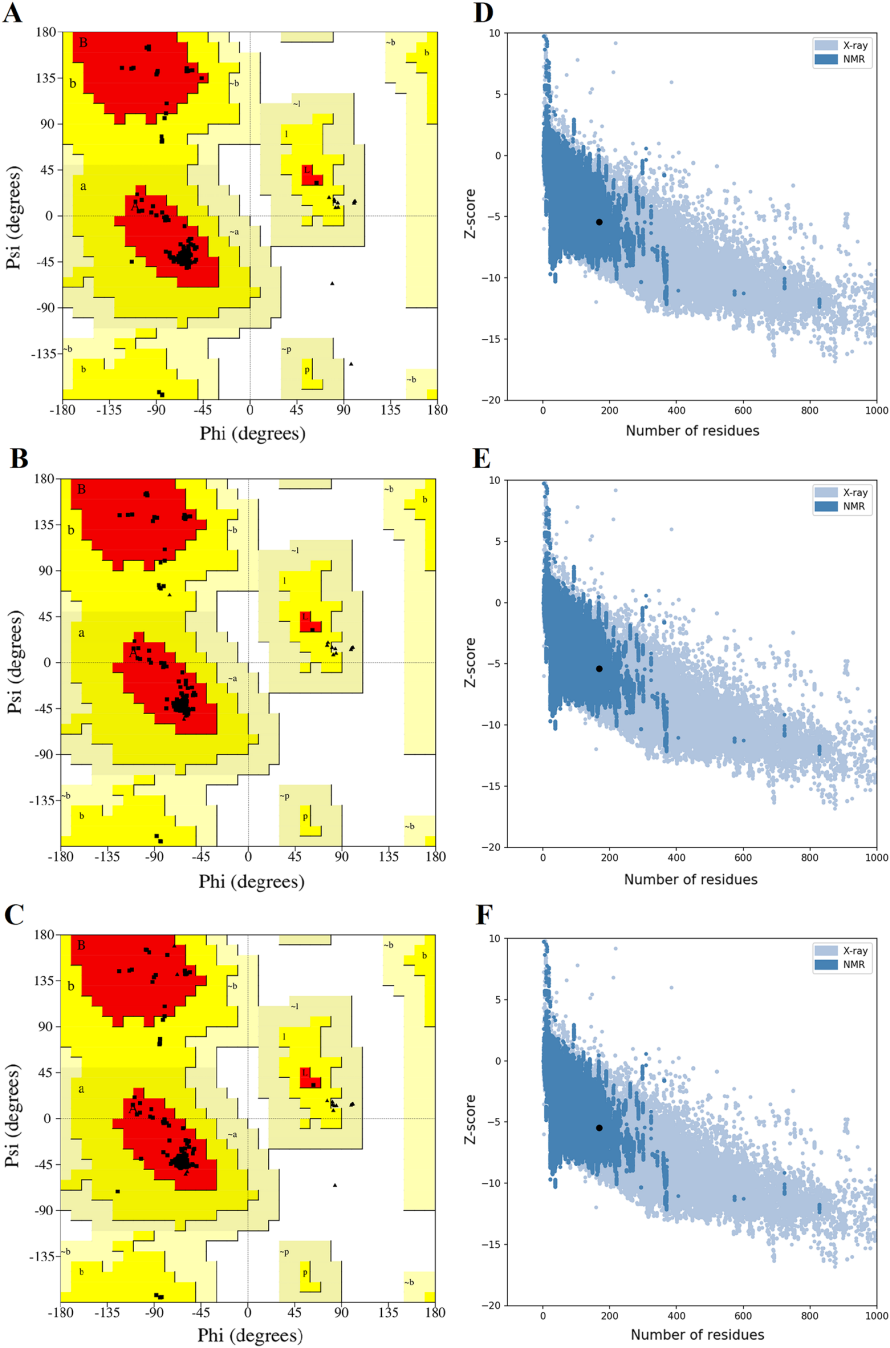
Ramachandran plot of the (**A**) nGLP1-DARPin, (**B**) mGLP1-DARPin-1, and (**C**) mGLP1-DARPin-2 fusion proteins generated by the PROCHECK program. The regions in the plots are labeled as follows: [A, B, L]: residue in most favored regions (red), [a, b, l, p]: residue in additional allowed regions (yellow), [~ a, ~ b, ~ l, ~ p]: residue in generously allowed regions (beige), the disallowed region is in white, glycine residues were shown as triangles; The majority of residues in the core and allowed regions are due to the stereochemical quality of the protein structure; The ProSA result of the engineered fusion proteins showed a Z-score of −5.45, −5.39 and −5.46 (black dot) for (**D**) nGLP1-DARPin, (**E**) mGLP1-DARPin-1, and (**F**) mGLP1-DARPin-2 fusion proteins, respectively, which was inside the range characteristic for native proteins.

The trRosetta-predicted 3D structures of the fusion proteins were assessed by the Ramachandran plot, ERRAT, and ProSA Web server. The Ramachandran plot showed that the proportion of residues in three engineered fusion protein models in the favored region was 94.00% and 6.00% in the allowed region. (Fig. 3A–C). The results of the Ramachandran plot indicated that the overall quality of the predicted models of the engineered fusion proteins is sufficiently good. The overall quality factors of the refined models on the ERRAT server were 99.375% for nGLP1-DARPin and mGLP1-DARPin-1, and 100% for mGLP1-DARPin-2 (Fig. S3). According to the ERRAT results, the final refined models have the highest overall quality factor. And also, the ProSA-calculated Z-scores for nGLP1-DARPin, mGLP1-DARPin-1, and mGLP1-DARPin-2 were −5.45, 5.39, and −5.46, respectively, which were inside a characteristic range for native proteins (Fig. 3D–F).

### Molecular dynamics

**Figure 4:**
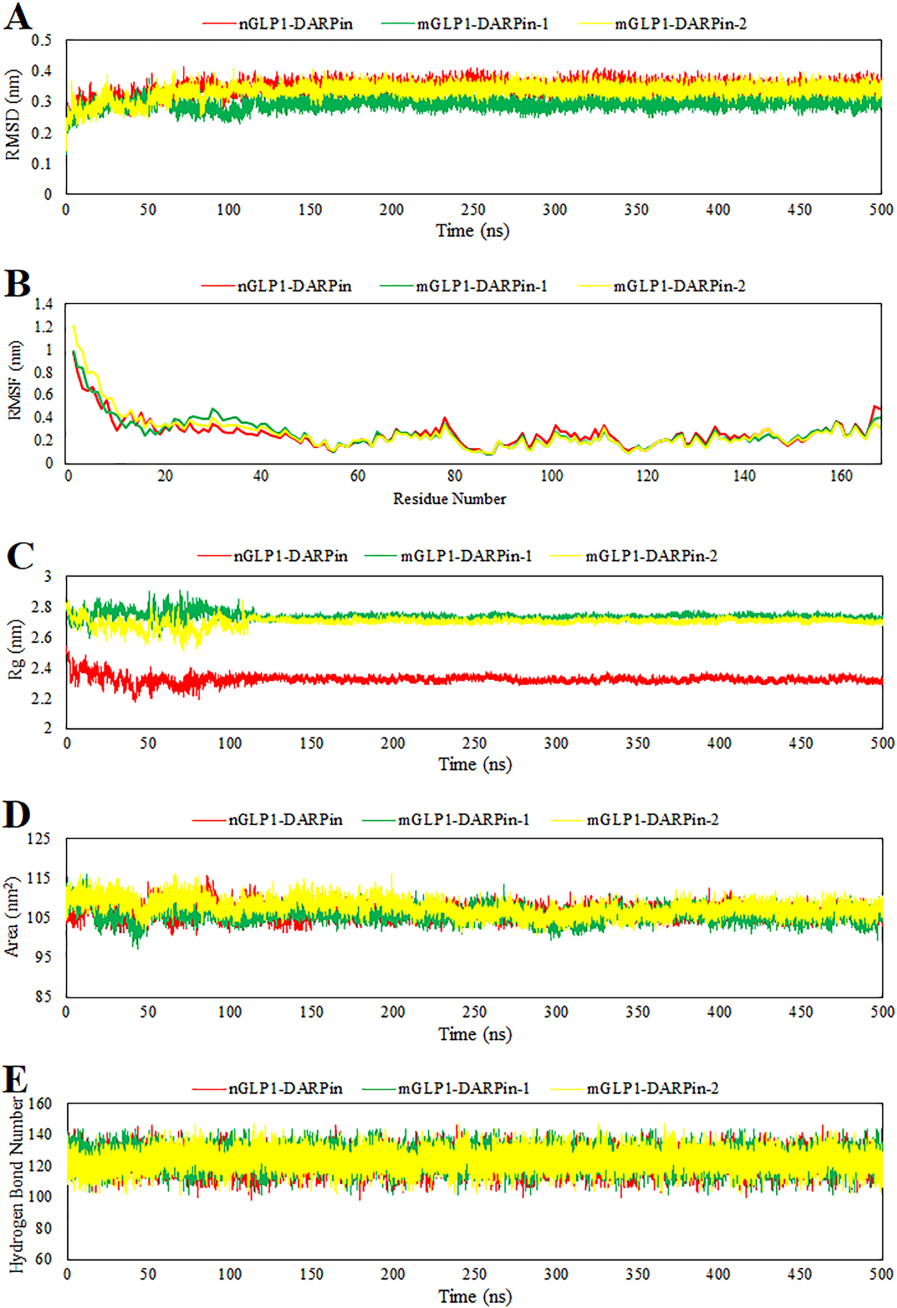
(**A**) Root mean square deviation, (**B**) root mean square fluctuation, (**C**) gyration radius graph, (**D**) solvent accessible surface area (SASA), and (**E**) hydrogen bond formation and deformation for MD simulation of the predicted structure of the nGLP1-DARPin (red), mGLP1-DARPin-1 (green), and mGLP1-DARPin-2 (yellow) fusion proteins.

We performed three replica molecular dynamic simulations for 500 ns to evaluate the dynamic behavior of the engineered fusion proteins as described in the methods. The simulation bridges the gap between theory and experimentation. The theory was tested through simulation using computer-generated models that provided insight into the utmost stability of complex molecules and the potential for strong interactions between molecules^39,40^. The GROMACS program was used to assess the stability of the engineered fusion proteins, and the PyMOL package was used to visualize their structure. The MD simulation output data were evaluated based on RMSD^41^, RMSF, SASA, Gyration radius^42^, and the average number of H-bonds, with the results summarized in Table 2 and Fig. 4.

**Table 2:** Results of the last 500 ns molecular dynamic simulation for the fusion proteins.

Parameters	nGLP1-DARPin	mGLP1-DARPin-1	mGLP1-DARPin-2
Protein backbone RMSD	0.34 ± 0.022	0.29 ± 0.017	0.33 ± 0.024
Protein backbone RMSF	0.264 ± 0.121	0.265 ± 0.133	0.268 ± 0.159
Gyration radius graph	2.32 ± 0.025	2.73 ± 0.023	2.70 ± 0.027
Solvent Accessible Surface Area	106.56 ± 1.54	105.93 ± 1.75	107.64 ± 2.02
Hydrogen bond formation/deformation	122.53 ± 5.73	124.84 ± 5.85	123.79 ± 5.80

Data represent mean ± SD.

*RMSD* root mean square deviation, *RMSF* root mean square fluctuation; *Rg* radius of gyration.

The average distance between the atoms that make up a protein's backbone relative to its initial structure is measured by RMSD. The RMSD plots for predicted models of the fusion proteins reached a constant value after 130 ns and flattened with a minimal deviation for the rest of the simulation time (Fig. 4A). The RMSD mean value of the nGLP1-DARPin fusion protein was 3.4 Å, which was slightly higher than for two fusion proteins containing modified GLP-1 (Fig. 4A, Table 2). This indicates that the stability of the mutant systems is relatively higher than that of the native GLP-1-containing system.

The root mean square fluctuation (RMSF) of Cα atoms in engineered fusion proteins was determined using final trajectories to analyze the mean atomistic motions of distinct residues. More flexibility (more conformational fluctuation) is indicated by higher RMSF values, whereas less fluctuation in the structure is indicated by lower RMSF values. Comparing the fluctuations of the RMSF value between different fusion protein structures, as seen in Fig. 4B, we found that the GLP-1 moiety of the fusion protein is more flexible than other regions of the fusion protein. Among the most flexible regions, we observed more flexibility in modified residues of the mGLP1-DARPin-1 and mGLP1-DARPin-2 fusion proteins compared with the fusion protein containing native GLP-1.

The radius of gyration indicates the level of compaction in the structure and the folding properties of the protein. The gyration radius reached the plateau after 130 ns and remained constant over the timeframe with an average value of 2.32, 2.73, and 2.7 nm for nGLP1-DARPin, mGLP1-DARPin-1, and mGLP1-DARPin-2, respectively, which signifies that all systems remained compact across the 500 ns MD simulation (Fig. 4C). The higher Rg values of the mGLP1-DARPin-1 and mGLP1-DARPin-2 fusion proteins than the nGLP1-DARPin indicate loose packing of the protein structure in the mutant systems, which means more flexible conformation. In theory, changes in protein accessibility to solvents can be determined by computing the solvent-accessible surface area (SASA). The SASA plots of all engineered fusion proteins are shown in Fig. 4D, and their average quantities are depicted in Table 2. During the simulations, the SASA of the proteins would naturally increase as hydration of the hydrophobic core occurred during unfolding, disrupting hydrophobic interactions among non-polar residues. The fusion protein's SASA plot revealed slightly diminishing trends, indicating reduced exposure of the hydrophobic core to solvation as unfolding progressed, making the proteins more stable (Fig. 4D). The average number of H-bonds in each frame over time corresponds to the number of hydrogen bonds formed or broken throughout the molecular simulation. Figure 4E displayed a stable fluctuation in the pattern of the number of hydrogen bonds formed in all systems, and this number was practically constant during the 500 ns of MD simulation in all systems, suggesting the stability of the molecule structures. The accuracy of our engineered fusion protein structures was validated by the fusion protein simulation results analysis.

**Figure 5:**
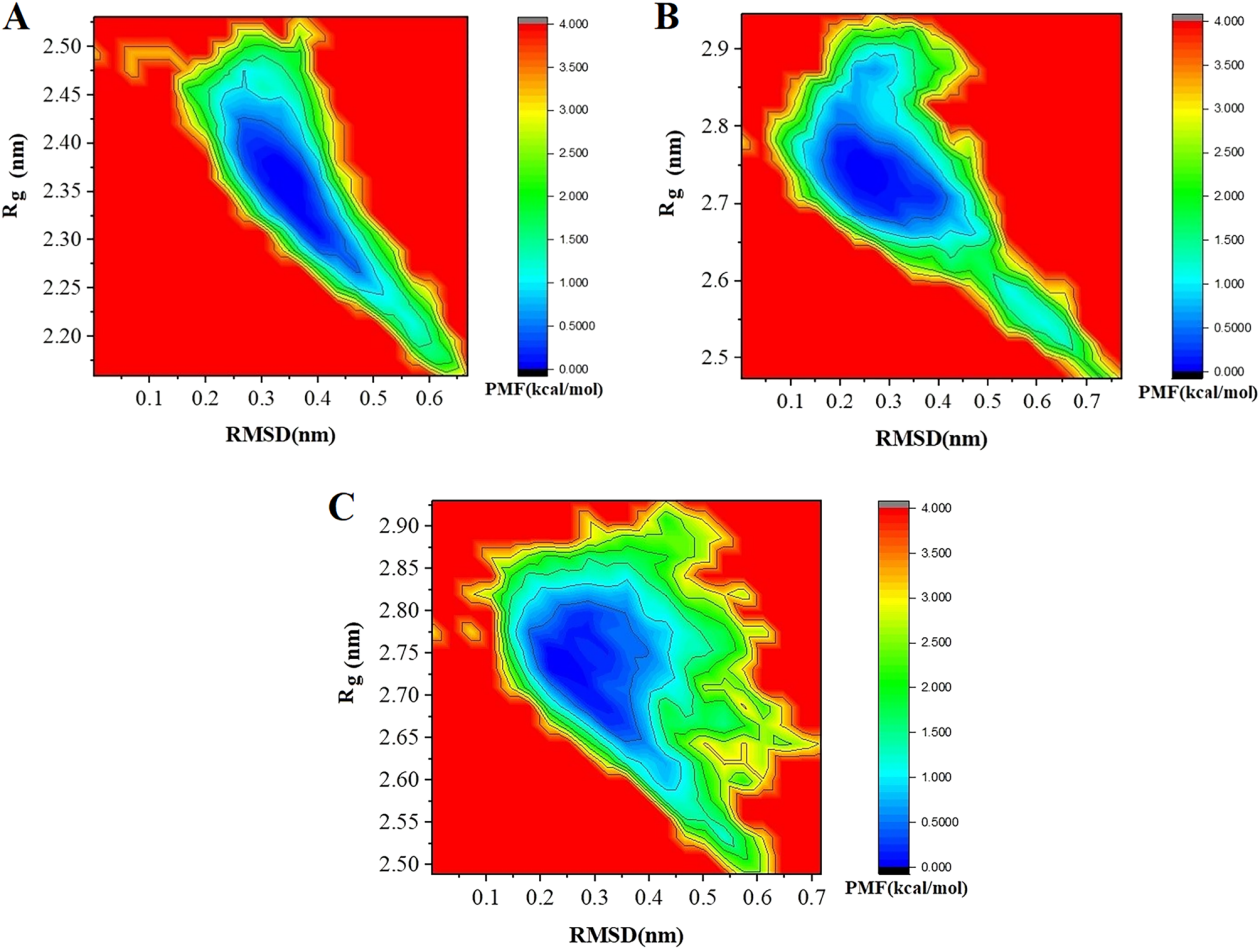
2D-PMF profiles regarding the RMSD and Rg of (**A**) nGLP1-DARPin, (**B**) mGLP1-DARPin-1, and (**C**) mGLP1-DARPin-2.

The calculation of the free energy landscape is based on the potential of mean force (PMF), which is defined by ∆G = −*k*_*B*_Tlnρ(x, y) where temperature is represented by T and *k*_*B*_ is the Boltzmann constant. Similarly, x and y represent Rg and RMSD, respectively. In Fig. 5, 2D-PMF profiles are displayed. Our findings showed that for the nGLP1-DARPin, mGLP1-DARPin-1, and mGLP1-DARPin-2, the most stable configurations appeared approximately at (RMSD = 0.35 nm and Rg = 2.35 nm), (RMSD = 0.25 nm and Rg = 2.74 nm), and (RMSD = 0.25 nm and Rg = 2.75 nm), respectively. Based on the results of the MF profile computation, it can be concluded that the changed residues of the mGLP1-DARPin-1 and mGLP1-DARPin-2 fusion proteins are more stable than those of the native protein.

### Protein–protein interaction of fusion proteins

MD results indicated that all engineered fusion proteins may retain their biological activity. Using molecular docking, we aimed to examine whether each moiety of the engineered fusion proteins could bind to its receptor. The frame with the lowest RMSD value with the average structure from the last 50 ns of each trajectory from all replicas of MD simulations of fusion proteins was selected for further docking steps. We used the crystallography structures of the extracellular domain of the GLP-1 receptor (PDB ID: 3IOL) and human serum albumin (PDB ID: 1AO6) to enter the docking steps. We used the ClusPro 2.0 online server for protein–protein docking analysis and the PRODIGY server to calculate binding free energy for each engineered fusion protein. As a control, GLP-1 (PDB ID: 1D0R) with the GLP-1 receptor docking analyses, and the HSA-binding DARPin (trRosetta predicted structure) with human serum albumin have been conducted.

**Figure 6:**
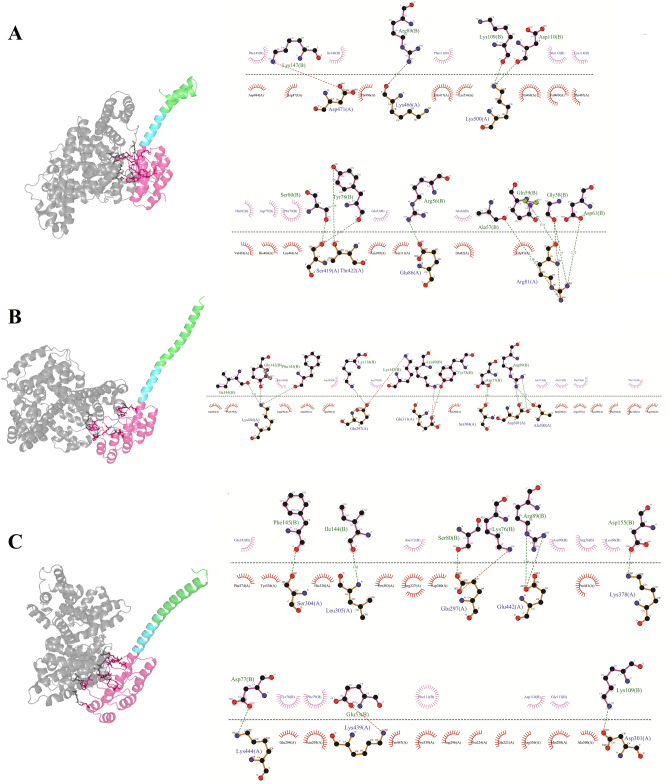
Interaction between (**A**) nGLP1-DARPin, (**B**) mGLP1-DARPin-1, and mGLP1-DARPin-2 fusion proteins with human serum albumin (gray). GLP-1 is depicted in green, while the linker is shown in cyan. HSA-binding DARPin is colored hot pink.

**Figure 7:**
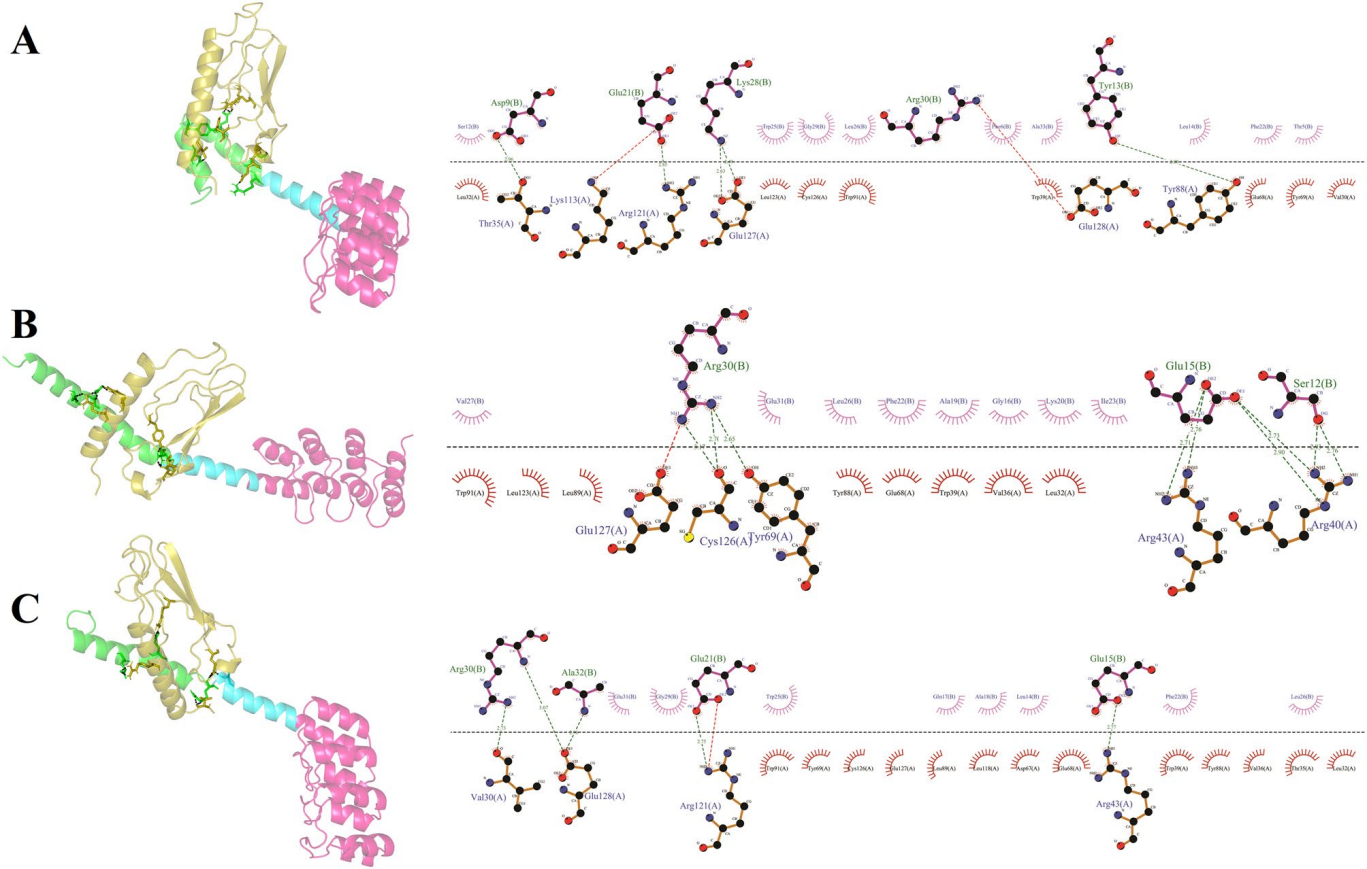
Interaction between (**A**) nGLP1-DARPin, (**B**) mGLP1-DARPin-1, and mGLP1-DARPin-2 fusion proteins with the extracellular domain of the GLP-1 receptor (olive). GLP-1 is depicted in green, while the linker is shown in cyan. HSA-binding DARPin is colored hot pink.

We used the docking energy score as the selection criterion for the best-docked complex, so that the lower the binding energy scores, the better the binding affinity between the two proteins. The results indicated that all engineered fusion proteins showed a relatively high docking score, suggesting that they may have lower energy constraints for binding (Table 3). Regarding binding affinity, docking analysis of engineered fusion proteins with HSA showed higher quantities than the GLP-1 receptor, suggesting their highest potential for binding to HSA (Table 3). This can be supported by the highest number of hydrogen bonds in the DARPin moiety of fusion proteins established with HSA (Fig. 6). In addition, among the fusion proteins, nGLP1-DARPin had a higher binding affinity to HSA (ΔG = −9.2 kcal mol^−1^) compared to the mGLP1-DARPin-1 (ΔG = −8.1 kcal mol^−1^) and mGLP1-DARPin-2 (ΔG = −8.3 kcal mol^−1^) fusion proteins, which is comparable with the individual DARPin complexed with HSA (ΔG = −9.6 kcal mol^−1^). In the docking analysis with the GLP-1 receptor, it can be seen that the nGLP1-DARPin fusion protein had a higher binding affinity to the GLP-1 receptor (ΔG = −6.1 kcal mol^−1^) compared with the mGLP1-DARPin-1 (ΔG = −5.5 kcal mol^−1^) and mGLP1-DARPin-2 (ΔG = −5.8 kcal mol^−1^) fusion proteins (Table 3, Fig. 7). Regarding the modified GLP-1 containing fusion proteins, mGLP1-DARPin-2 with Aln8Gly, Lys26Gn, and Lys34-Asp substitutions had a higher binding affinity to the HSA and GLP-1 receptor than GLP1-DARPin-1 with an Aln8Gly substitution. The findings demonstrated that the interactions between GLP-1/GLP-1R and HSA-DARPin/HSA persisted even after the amino acid substitution in GLP-1 and the formation of the fusion protein.

**Table 3:** Docking scores and ΔG of protein–protein complexes obtained from the Cluspro and PRODIGY servers.

Protein–Protein Complex	Docking score	ΔG (kcal mol^−1^)	Dissociation constant (M)
DARPin::HAS	−732.3	−9.6	1.7e−07
nGLP1-DARPin::HAS	−752.3	−9.2	1.8e−07
mGLP1-DARPin-1::HAS	−746.2	−8.1	3.9e−06
mGLP1-DARPin-2::HAS	−810.6	−8.6	1.7e−07
GLP-1::GLP-1 receptor	−820.6	−6.8	1.6e−05
nGLP1-DARPin::GLP-1 receptor	−897.4	−6.1	3.2e−06
mGLP1-DARPin-1::GLP-1 receptor	−821.9	−5.5	3.4e−05
mGLP1-DARPin-2::GLP-1 receptor	−852.3	−5.8	1.2e−05

**Figure 8:**
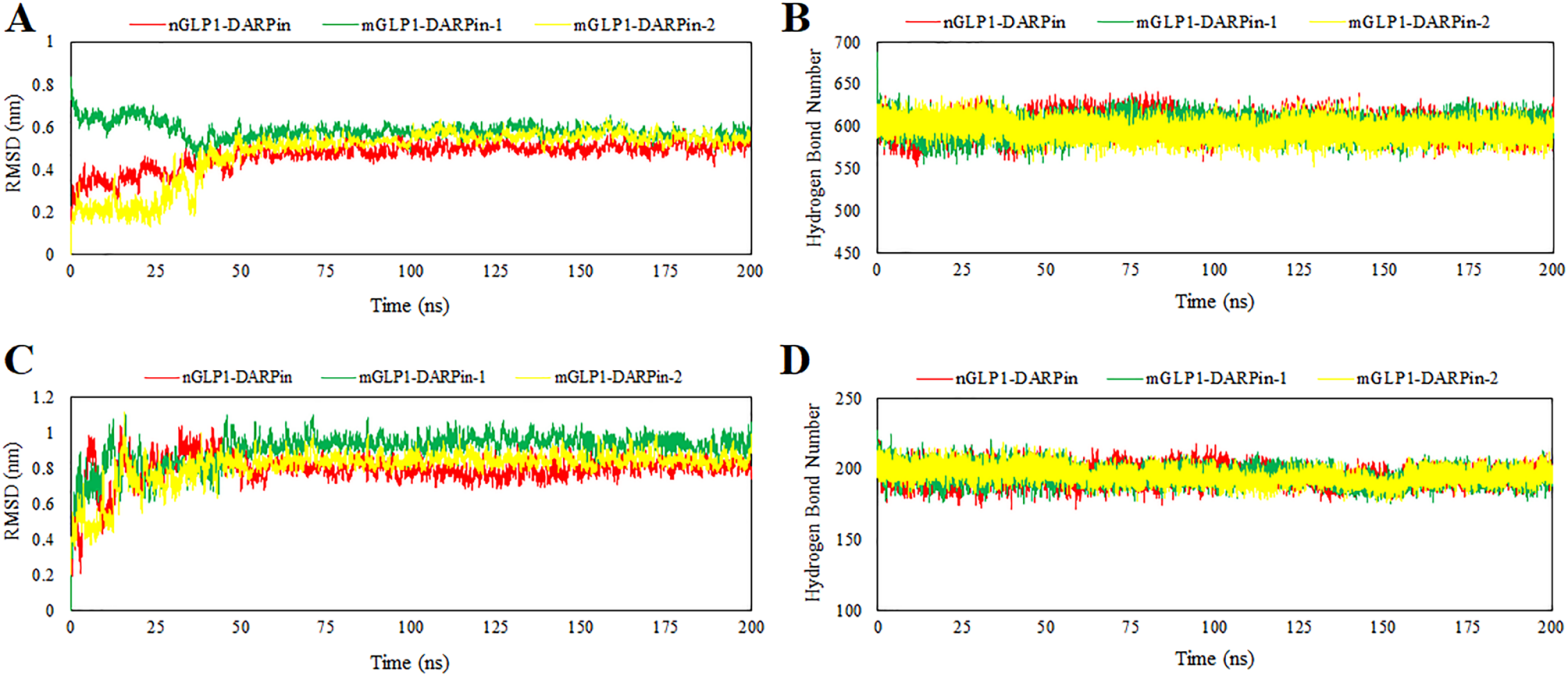
Carbon backbone RMSD profiles of the fusion proteins in complex with (**A**) human serum albumin and (**B**) the extracellular domain of the GLP-1 receptor; and the average number of H-bonds formed by each fusion protein with (**C**) human serum albumin and (**D**) the ECD of the GLP-1R during 200 ns MD simulations.

The complexes with a higher docking score were selected and molecular dynamically simulated using GROMACS 2019.2 software. The RMSD, representing structural variations during the simulation period compared to the time zero state, was obtained. The RMSD plot of the fusion proteins complex with HSA demonstrates that all three systems reached a stable state after 50 ns of the MD simulation. No significant fluctuations were observed for the rest of the simulation period, with average RMSD values of 0.47 ± 0.06, 0.58 ± 0.04, and 0.48 ± 0.12 for the nGLP1-DARPin, mGLP1-DARPin-1, and mGLP1-DARPin-2 complexed with HSA, respectively (Fig. 8A). The number of H-bonds formed between fusion proteins and the receptors at each step of the simulation was counted to ensure that the binding interaction was stable throughout the MD simulation. The total number of H-bonds forming in all three fusion proteins complexed with HSA was about 600, which remained consistent over the simulation time (Fig. 8B).

The RMSD plot analysis of fusion proteins complexed with GLP-1 receptor during 200 ns of the MD simulation showed that all three docked complexes stabilized after 50 ns and remained stable throughout the rest of the simulation time with RMSD values of 0.8 ± 0.08, 0.9 ± 0.09, and 0.81 ± 0.1 for nGLP1-DARPin, mGLP1-DARPin-1, and mGLP1-DARPin-2 complex with GLP-1R, respectively (Fig. 8C). Regarding H-bonds, internal hydrogen bonds in all three fusion proteins complexed with the GLP-1 receptor were about 195 and remained stable throughout the simulation with no obvious fluctuations (Fig. 8D).

## Discussion

Our research aimed to design and perform computational analysis of the fusion proteins with high affinity and specificity for human serum albumin and a protease-resistant GLP-1 peptide to produce potent GLP-1 agonists with an extended plasma half-life for the treatment of type 2 diabetes. The rapid enzymatic degradation and short plasma half-life of GLP-1 (about 2 min) limit its therapeutic use. To address this limitation, research has concentrated on many strategies, including identifying and developing DPP-IV inhibitors^43,44^, altering the structural makeup of GLP-1, searching for structural analogues that are more resistant to DPP-IV cleavage^45^, and increasing its hydrodynamic radius (*R*h) above the glomerular filtration cutoff by conjugating it to proteins such as transferrin^46^, albumin^47^, and elastin-like polypeptides^48^. Since the fusion of an albumin-binding moiety to proteins instead of directly fusing biologics to albumin has several advantages in terms of production cost and safety^49^, the use of a binding protein known as DARPins has been decided upon in this work. DARPins can selectively bind any given target protein with high affinity^30^, and HSA can be specifically targeted by albumin-binding DARPin too. As demonstrated by MP0250^28^, the therapeutic protein's half-lives can be significantly extended by genetically fusing non-HSA-binding DARPins or other therapeutic proteins to albumin-binding DARPins. The many benefits that DARPins possess make them the perfect scaffold for the development of protein drugs, including high thermal stability, with denaturation midpoints from 66 to 95 °C, and high solubility in solution, up to 100 mg/mL^30,50^. In incubation with human serum (ex vivo), no proteolytic digestion was observed, indicating high stability for potential drug use. *E. coli*, yeast, and plants can all express DARPins at high levels in the soluble form^31,33,34^, and based on chemical characterization, DARPins are predicted to be non-immunogenic^51^. The albumin-binding DARPin domain demonstrated nanomolar affinities for humans, cynomolgus monkeys, mice, and rats’ serum albumin at pH 7.4 or 6.0. Because of this broad species selectivity, half-life data can be extrapolated from mice to humans^28^. Due to these characteristics, albumin-binding DARPins are a potential substitute scaffold for extending the half-life of therapeutic proteins and peptides.

Based on these studies and the functional form of native GLP-1 (7–36), chimeric proteins as potent GLP-1 agonists with an extended plasma half-life were engineered using a native or two modified human GLP-1 (mGLP-1) that were resistant to DPP-IV or both DPP-IV and trypsin cleavage and fused to a DARPin with high affinity and specificity for HSA. To create the modified GLP-1, the enzyme-sensitive Ala at position 8 and Lys at positions 26 and 34 were substituted by Gly, Gln, and Asp, respectively. It has been demonstrated that these structural alterations exert complete resistance to GLP-1 cleavage caused by DPP-IV and trypsin degradation^52–55^. In a recent study, Tan and coworkers engineered and expressed two GLP-1 analogues by genetically fusing a modified GLP-1 (Ala8Gly) to one or two tandem HSA-binding DARPins through a (GGGGS)_3_ flexible linker. Their findings revealed that GLP-2DARPin, which binds two HSA molecules, had a half-life roughly three times longer than GLP-DARPin (52.3 h versus 18.0 h). The bioactivity results, however, showed that GLP-DARPin had a stronger blood glucose-lowering effect than GLP-2DARPin. In addition, according to the oral glucose tolerance tests, GLP-DARPin significantly lowered blood glucose levels for at least 48 h, whereas GLP-2DARPin did so for only 24 h^56^. In another recent study, Xia et al. designed and expressed four injectable long-acting GLP-1R agonists in a bacterial expression system by genetically fusing Exendin-4 to the N-terminus of HSA-binding DARPin via linkers of different types and lengths. Their results demonstrated that Ex-DARPin fusion proteins were substantially stable, resulting in incomplete denaturation even at 80 °C. The in vitro bioactivity results showed that Ex-DARPin fusion proteins could bind to HSA, activate GLP-1R, and have a longer half-life than native Ex^57^.

We engineered our chimeric proteins with a rigid helical linker because previous research with albiglutide demonstrated that albumin's proximity to GLP-1 can significantly reduce its activity. That’s why albiglutide contains two copies of GLP1 in tandem, with the second copy fused to HSA, which serves as a steric spacer^36^. The selection of a linker is a crucial step in the design process of a fusion protein, especially in preserving the biological activity of the domains. We chose (EAAAK)_3_ rigid helical linkers to work because we believe their rigidity allows for the separation of the protein domains and improves the functionality of individual domains^58,59^ when compared to flexible linkers, which appear to be suitable for linking normally connected domains in a single genetic construct, such as antibody domains in scFvs^60^. With a GLP-1 fusion protein containing an albumin binding domain, Yousefpour and colleagues^61^ showed that the longer length and rigidity of the helical linker effectively kept the albumin, which is bound to the albumin binding domain, far enough from GLP1 to prevent it from interfering with its binding to the GLP1-R. In contrast, the shorter and more flexible linker does not completely remove the steric hindrance, leading to decreased receptor binding.

Bioinformatics approaches can result in significant time, cost, and failure reductions in experimental attempts. Using various bioinformatics tools and servers, we investigated the structural, evolutionary, and physicochemical characteristics of the engineered fusion proteins. The engineered constructs were 168 amino acids in length, with native or modified GLP-1 ranging from 1 to 30. The linker sequence was amino acids 31–45 (15 aa), and the HSA-binding DARPin was 46–168 (123 aa). Because the N-terminus of GLP-1 is critical to its biological function, we engineered the fusion proteins to leave the N-terminus of GLP-1 free. ProtParam was used to determine the primary structural features of the engineered fusion proteins. The high extinction coefficient, low instability index, and high aliphatic index of engineered fusion proteins indicate they are stable over a wide temperature range. SOPMA and PORTER, which perform a probabilistic assessment for all amino acids together, assisted us in making our secondary structure prediction. Alpha-helices make up a large portion of our fusion proteins. Furthermore, Protein-sol revealed that our engineered fusion constructs were more soluble than the soluble *E. coli* proteins in the experimental dataset. Following that, in silico analysis was performed to ensure that each domain in the engineered fusion proteins was folded correctly. The details of the 3D structure of our engineered fusion proteins can be used to study protein function, dynamics, and interaction with receptors. TrRosseta was used for the 3D modeling of our fusion constructs, and structure validation was performed using the Ramachandran plot, ProSA-web, and ERRAT tools to detect errors in the 3D modeled structures of the fusion constructs. Further validation of the engineered fusion protein's modeled tertiary structure was performed using the GROMACS MD simulation package for 500 ns in a water environment. After simulation, molecular docking studies were used to study the stable interactions of the engineered fusion proteins against the GLP-1 receptor and human serum albumin. More importantly, docking results demonstrated that the engineered fusion proteins can recognize and bind both GLP-1R and HSA with high affinity.

## Conclusion

In this study, we designed long-acting chimeric proteins as GLP-1 receptor agonists for the treatment of type 2 diabetes mellitus and evaluated them through bioinformatics approaches. The mGLP1-DARPin-1 fusion protein that was more resistant to DPP-IV cleavage can be used as a long-lasting injectable form of GLP-1, and the mGLP1-DARPin-2 fusion protein that was resistant to both DPP-IV and trypsin cleavage can be used as a candidate for oral delivery of GLP-1 bioencapsulated in plant cells. According to the in silico results, the engineered fusion proteins can identify and bind to the two target proteins (GLP-1R and HSA). Our study's methodology facilitates rapid analysis of engineered chimeric constructs prior to starting laboratory experiments with recombinant fusion proteins. Since all software and servers are available for free, it should go without saying that this process is quick, affordable, and easy—especially for novice users in this field. In conclusion, as we demonstrate here, the fusion of peptide therapeutics to an albumin-binding DARPin, if engineered properly, is a straightforward and modular protein engineering strategy to enhance the therapeutic efficacy of short-circulating biologics. Although the present computational study provides three potential candidates as long-acting GLP-1 agonists, an experimental process needs to be established, a matter which is the theme of our ongoing study.

## Methods

The current study involves the fusion of a native or mutant GLP-1 (30 residues) with an HSA-binding DARPin (123 residues) via a rigid helical linker (15 residues) to create fusion proteins (168 residues) resistant to DPP-IV and, or trypsin with extended half-lives using structural modeling, which are subsequently characterized using MD simulation and molecular docking analyses.

### Sequences retrieval and fusion proteins design

The amino acid sequence for the DARPin with a high affinity for human serum albumin was obtained from US Patent 2016/9458211 B1^62^. The GLP-1 molecule, which originally consisted of 30 amino acids from human GLP-1 (7–36), was modified with the substitution of alanine (Aln) by glycine (Gly) at position 8 to prevent DPP-IV recognition and degradation and with glutamine (Gln) and aspartic acid (Asp) instead of lysin (Lys) at positions 26 and 34, respectively, to inhibit trypsin digestion. Based on previous studies, the N-terminal of GLP-1 is important for its biological function; therefore, we engineered the fusion constructs to free the N-terminal of GLP-1. The native or two modified GLP-1 (mGLP-1) with Ala8Gly substitution or Ala8Gly, Lys26Gln, and Lys34Asp substitutions were genetically fused to the N-terminus of HSA-binding DARPin through a rigid helical linker (EAAAK)_3_ to create the fusion proteins, denoted as nGLP1-DARPin, mGLP1-DARPin-1, and mGLP1-DARPin-2, respectively. Rigid linkers are used when there is a requirement to preserve the spatial distance between the domains and their independent functions.

### Primary structure properties of fusion proteins

Physical and chemical characteristics of engineered fusion proteins, including all molecular weight, molecular formula, theoretical isoelectric point (pI), the total number of positive and negatively charged residues in the sequence, extinction coefficient (E), instability index (II), aliphatic index (AI), and grand average hydropathy (GRAVY), were attained using the Expasy ProtParam server^63^. The instability index estimates a protein's in vitro stability, so the instability index must be below 40 for a protein to be considered stable. A protein aliphatic index is described as the relative volume occupied by aliphatic side chains (leucine, isoleucine, valine, and alanine) and is viewed as a favorable factor for the rise of thermostability in globular proteins. The GRAVY score is calculated by adding all of the amino acid hydropathy scores and dividing them by the number of residues in the sequence.

### Prediction of secondary structure

The engineered fusion proteins’ secondary structures, including the regions lacking regular shape, sections with low complexity, the proportion of random coils, extended strands, and alpha helices, were predicted by the SOPMA^64^ and PORTER^65^ servers.

### Three-dimensional model prediction

The 3D structure of the engineered fusion proteins was modeled using the trRosetta web server^66^. The trRosetta algorithm predicts protein structures quickly and precisely. The raw amino acid sequences of the engineered fusion proteins were submitted to the trRosetta server in FASTA format. It builds the 3D structure using a restricted Rosetta and direct energy minimization. When the conditions of Confidence > 0.6, with an E-value of 0.001, and Coverage > 0.3 are met, a template is employed for subsequent prediction. The TM-score of the predicted models is calculated using the probability of the top anticipated distance and the convergence of the top models. The TM-score is a number between 0 and 1, with a value greater than 0.5, indicating that the model’s topology was predicted correctly. The trRosetta results produced five top models for each entry, of which the one with the greatest estimated TM-score (e-TM) represented the best model and was selected for this study. The predicted 3D structures were visualized by PyMOL Version 2.3.2_81 (the PyMOL Molecular Graphics System).

### Tertiary structure validation

Procheck Ramachandran Plot v.3.5.4^67^ and ERRAT^68^ of the SAVES V6.0 web tool and ProSA-Web^69^ were used for structural evaluation and stereochemical analyses. The Ramachandran plot showed the depiction and distribution of the residues in favored, allowed, and outlier regions and was used to determine the validity and quality of protein models. In the predicted structure, the ERRAT server evaluated the statistics of non-bonded interactions among various atom types by comparison with statistics from highly refined structures, and its higher scores indicate higher quality. The ProSA Z-score estimates the total energy of the structure from an energy distribution produced from random conformations and represents the overall model quality.

### Protein solubility prediction

The Protein-Sol web server^70^ was used to estimate the solubility of engineered fusion proteins. It analyzes amino acid sequences and computes anticipated solubility and other attributes. For experimental solubility, since the population average for the experimental dataset (PopAvrSol) is 0.45, any scaled solubility value greater than 0.45 is predicted to be more soluble than the average soluble proteins of *E. coli*^71^. In contrast, any protein with a lower scaled solubility value is predicted to be less soluble. The toxic potential of engineered fusion proteins was predicted using the ToxDL^72^ web server. The ToxDL server is devised for deep learning prediction of toxic domains in protein structures.

### Molecular dynamics simulation

The GROMACS package, version 2019.2, was used to simulate the molecular dynamics of engineered fusion proteins^73^. For each fusion protein, the best model obtained from molecular modeling was subjected to MD simulation. The all-atom Charmm27 forcefield was selected for making topology files, and the structure was placed in a triclinic unit cell 1.0 nm from the box edge. The system was first solvated using a simple point charge (SPC) water model before being ionized and neutralized by Na^+^ and Cl¯ ions with a salt concentration of 0.15 M. Following that, a conjugate gradient algorithm with Steepest Descent (SD) minimization was used to minimize energy on a model that had already been predicted. Then the position restraint simulation was conducted under an NVT (constant number, volume, and temperature) ensemble heated to 300 °K at 100 ps and an NPT (constant number, pressure, and temperature) equilibrated to the 1 bar pressure at 100 ps. Finally, an unrestrained MD simulation was run for 500 ns on three replicas with coupled temperature (300 °K) and pressure (1 bar). Microsoft Office Excel was used to plot graphs and perform a comparative analysis of structural deviations based on extracting parameters from the MD trajectories such as Root Mean Square Deviation (RMSD), Root Mean Square Fluctuation (RMSF), Solvent-Accessible Surface Area (SASA), Radius of Gyration (Rg), and H-bond formation or deformation. The potential mean force (PMF) of the studied systems was computed using the MolAICal package^74,75^.

### Protein–protein interaction

Following molecular dynamics simulations, the fusion proteins were subjected to molecular docking simulations. The ClusPro 2.0 online server, which is a fully automated server engineered for protein–protein docking simulations, was used to undertake a molecular docking analysis of the fusion proteins against human serum albumin (HSA) and the extracellular domain of the GLP-1 receptor (ECD-GLP-1R)^76^. Protein–protein interactions are computed in three steps: rigid body docking using the Fast Fourier Transform (FFT) correlation method; clustering of generated structures based on RMSD to find the largest cluster that represents the likely models of the complex; and refinement of selected structures. ClusPro 2.0 requires PDB files of the proteins as input and provides four types of output models based on the scoring algorithms designated as balanced, electrostatic-favored, hydrophobic-favored, and Van der Waals electrostatic.

In our docking, we designated ECD-GLP-1R and HSA as the receptors and engineered fusion proteins as the ligands. To validate the docking studies, GLP-1 and HSA-binding DARPin were docked against the GLP-1 receptor and human serum albumin, respectively. The crystal structure of GLP-1 in complex with ECD-GLP-1R (PDB ID: 3IOL) was obtained from the RCSB data bank. To our knowledge, the crystallographic structure of HSA-binding DARPin has not been reported in the protein database. Therefore, we carried out computational modeling of HSA-binding DARPin by the MODELLER software 15.9^77^ based on the most similar available conformation (PDB ID: 4GRG). The complexes obtained after docking were used as input for further MD simulations to increase the accuracy of the resulting interactions. We carried out three replicas of MD simulations for 200 ns using the same procedure in the MD section described above, to study the stability of these docking complexes. MD output data were analyzed regarding RMSD and total numbers of H-bond formation or deformation. Subsequently, MD simulation trajectories were submitted to the PRODIGY server (PROtein binDIng enerGY prediction) to obtain free energy (ΔG)^78^, and studied for interaction analysis using LigPlot + v.2.2^79^. The PyMOL package was utilized for visual depiction.

### Supplementary Information

Supplementary Information.

## Data Availability

The data that support the findings of this study are available from the corresponding author upon reasonable request.

## Abbreviations

3D: Three-dimensional
DARPin: Designed ankyrin repeat protein
ECD: Extracellular domain
GLP-1: Glucagon like peptide-1
GR: Gyration radius
HSA: Human serum albumin
MD: Molecular dynamics
RMSD: Root mean square deviation
RMSF: Root mean square fluctuation
SASA: Solvent-accessible surface area
T2DM: Type 2 diabetes mellitus
