# Supplementary material for: Designing and computational analyzing of chimeric long-lasting GLP-1 receptor agonists for type 2 diabetes
Source: Sci Rep. 2023 Oct 18;13:17778. doi: 10.1038/s41598-023-45185-1 (PMC10584922; doi:10.1038/s41598-023-45185-1)
Supplement: Supplementary file 1 — Supplementary Information. [file 41598_2023_45185_MOESM1_ESM.docx]

**Designing and Computational Analyzing of Chimeric Long-lasting GLP-1 Receptor Agonists for Type 2 Diabetes**

**Maryam Ehsasatvatan^1^, Bahram Baghban Kohnehrouz^2*^**

^1^Department of Plant Breeding & Biotechnology, Faculty of Agriculture, University of Tabriz, Tabriz-51666, Iran. E-mail: [m.ehsasatvatan@yahoo.com](mailto:bahramrouz@yahoo.com). ORCID ID: orcid.org/ 0000-0001-8752-9203.

^2^Department of Plant Breeding & Biotechnology, Faculty of Agriculture, University of Tabriz, Tabriz-51666, Iran. E-mail: [bahramrouz@yahoo.com](mailto:bahramrouz@yahoo.com). ORCID ID: orcid.org/0000-0003-1558-7484.

*^*^Corresponding author***:** Bahram Baghban Kohnehrouz, Ph.D. in Molecular Biology and Biotechnology, Department of Plant Breeding & Biotechnology, Faculty of Agriculture, University of Tabriz, Tabriz 51666, Iran. Tel.: +98 41 33392031; Fax: +98 41 33356003; E-mail: [bahramrouz@yahoo.com](mailto:bahramrouz@yahoo.com); ORCID ID: orcid.org/0000-0003-1558-7484.

| Table S1 Toxicity assessment of engineered fusion proteins | | | |
| --- | --- | --- | --- |
| **Protein** | **ToxDL**  **Score** | **Result** | **Motif assessment** |
| nGLP1-DARPin | 0.014 | Non-toxic | 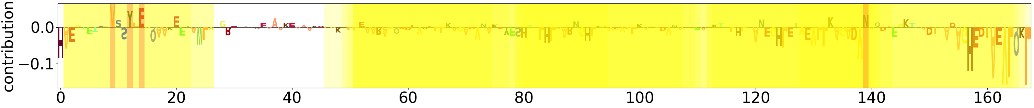 |
| mGLP1-DARPin-1 | 0.017 | Non-toxic | 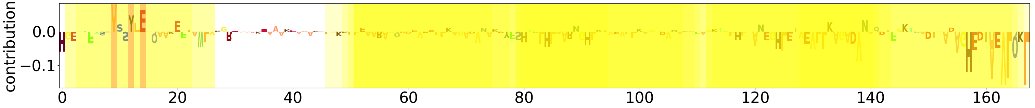 |
| GLP1-DARPin-2 | 0.05 | Non-toxic | 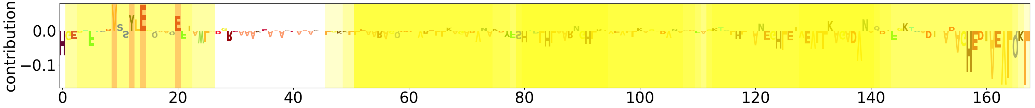 |


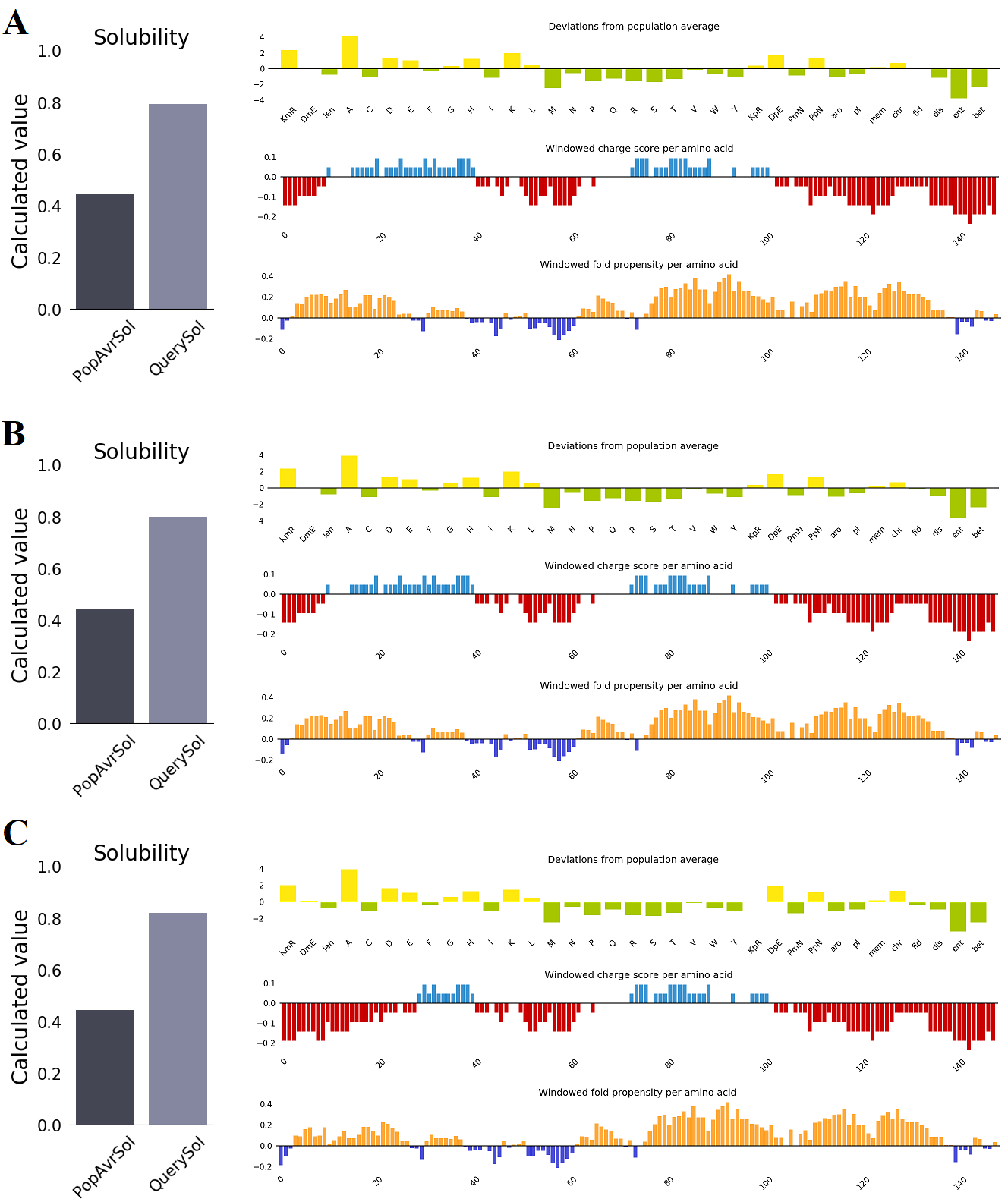


Figure S1 Predicted solubility of (A) nGLP1-DARPin, (B) mGLP1-DARPin-1, and (C) mGLP1-DARPin-2 fusion proteins predicted by Protein-Sol.


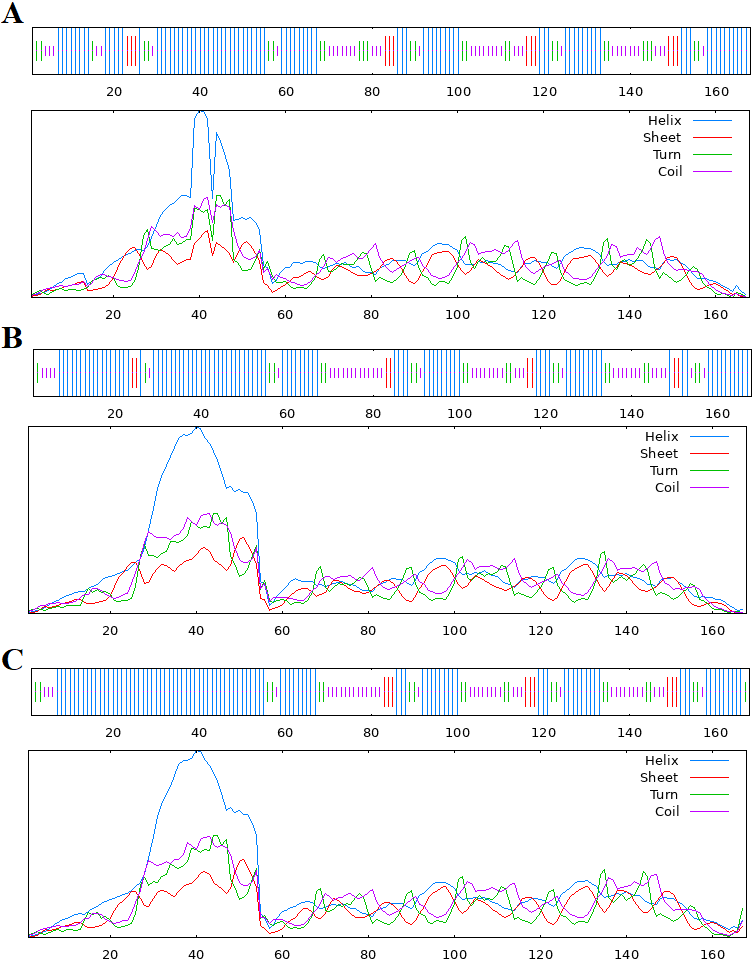


Figure S2 Graphical representation of secondary elements in the (A) nGLP1-DARPin, (B) mGLP1-DARPin-1, and (C) mGLP1-DARPin-2 fusion proteins predicted by SOPMA. Blue, purple, red and green indicate helices, extended strand, random coiled and turn composition, respectively.


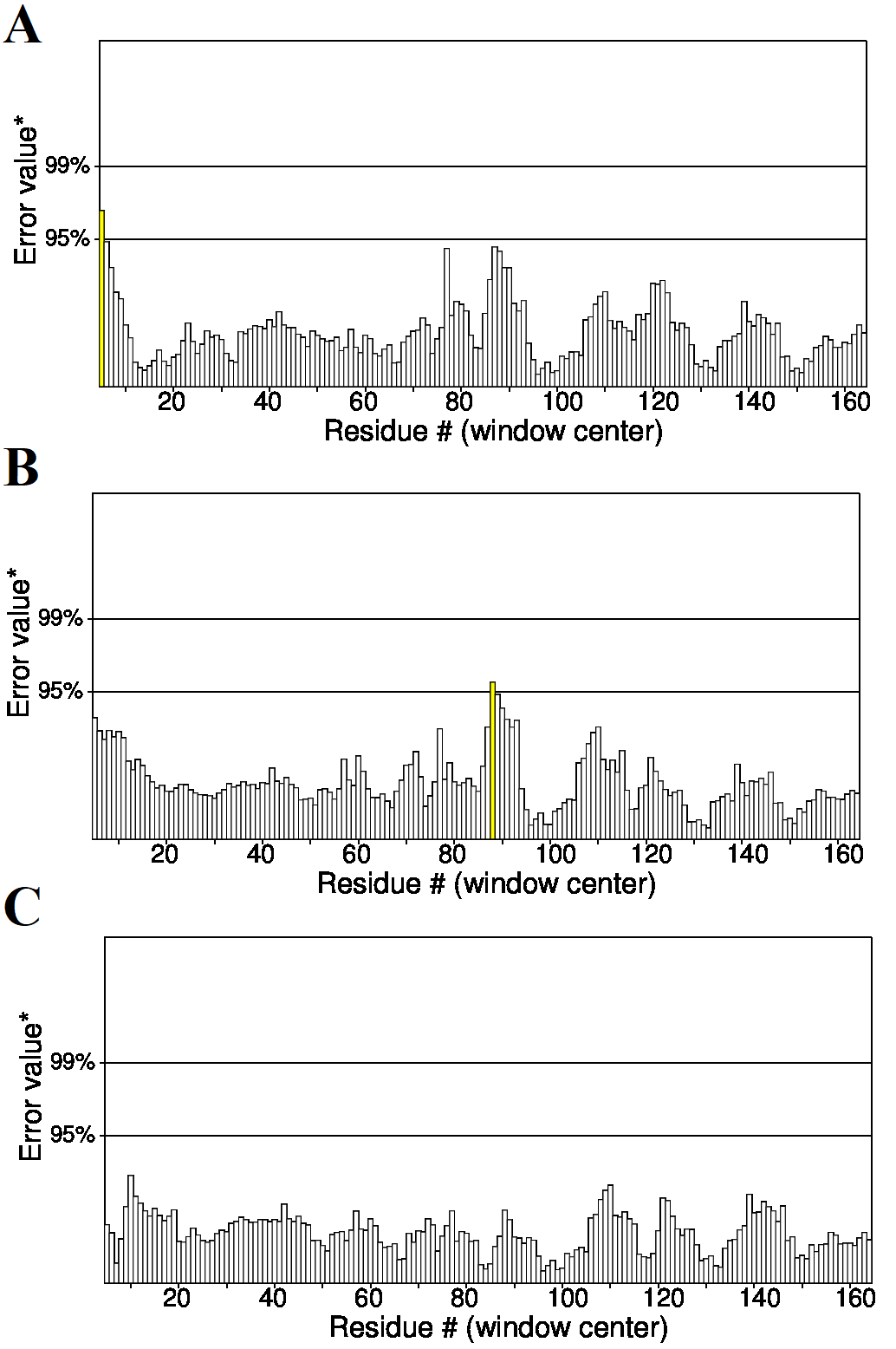


Figure S3 ERRAT result showing overall quality factor of (A) nGLP1-DARPin, (B) mGLP1-DARPin-1, and (C) mGLP1-DARPin-2 fusion proteins.
